# Cockroaches as a Source of High Bacterial Pathogens with Multidrug Resistant Strains in Gondar Town, Ethiopia

**DOI:** 10.1155/2016/2825056

**Published:** 2016-06-02

**Authors:** Feleke Moges, Setegn Eshetie, Mengistu Endris, Kahsay Huruy, Dagnachew Muluye, Tigist Feleke, Fisha G/Silassie, Getenet Ayalew, Raja Nagappan

**Affiliations:** ^1^Department of Microbiology, School of Biomedical and Laboratory Sciences, College of Medicine and Health Sciences, University of Gondar, P.O. Box 196, Gondar, Ethiopia; ^2^University of Gondar Hospital Laboratory, P.O. Box 196, Gondar, Ethiopia; ^3^Department of Internal Medicine, School of Medicine, College of Medicine and Health Sciences, University of Gondar, P.O. Box 196, Gondar, Ethiopia; ^4^Department of Biology, College of Natural and Computational Sciences, University of Gondar, P.O. Box 196, Gondar, Ethiopia

## Abstract

*Background*. Cockroaches are source of bacterial infections and this study was aimed to assess bacterial isolates and their antimicrobial profiles from cockroaches in Gondar town, Ethiopia.* Methods*. A total of 60 cockroaches were collected from March 1 to May 30, 2014, in Gondar town. Bacterial species were isolated from external and internal parts of cockroaches. Disk diffusion method was used to determine antibiotic susceptibility patterns. Data were entered and analyzed by using SPSS version 20; *P* values <0.005 were considered as statistically significant.* Results*. Of 181 identified bacteria species, 110 (60.8%) and 71 (39.2%) were identified from external and internal parts of cockroaches, respectively.* Klebsiella pneumoniae *32 (17.7%),* Escherichia coli *29 (16%), and* Citrobacter *spp. 27 (15%) were the predominant isolates. High resistance rate was observed to cotrimoxazole, 60 (33.1%), and least resistance rate was noted to ciprofloxacin, 2 (1.1%). Additionally, 116 (64.1%) of the isolates were MDR strains;* Salmonella *spp. were the leading MDR isolates (100%) followed by* Enterobacter *(90.5%) and* Shigella *spp. (76.9%).* Conclusion*. Cockroaches are the potential source of bacteria pathogens with multidrug resistant strains and hence effective preventive and control measures are required to minimize cockroach related infections.

## 1. Background

Cockroaches are insects with long antennae and legs, feeding by scavenging. Cockroaches are one of the most significant and objectionable pests found in apartments, homes, food-handling establishments, hospitals, and health care facilities worldwide. Indoor species, especially the German cockroach, exploit conditions associated with high-density human populations and impoverished living conditions [1]. Cockroaches consume garbage, rotting food, and even fecal waste of other roaches. They then transmit disease to your food, eating utensils, kitchen surfaces, and other areas around your home. They can easily contaminate food by leaving droppings which may contain bacteria that can cause food poisoning, fungi, and other pathogenic organisms [2–5]. Their nocturnal and filthy habits make them also ideal carriers of various pathogenic microorganisms [6, 7].

Various bacteria may simply be carried on the insect's cuticle or be ingested and, some time later, regurgitated or excreted. Moreover, several species of bacteria of public health significance have been isolated from, or have passed through, cockroaches (*Periplaneta americana*) and their digestive tract, such as* Staphylococcus aureus*,* Streptococcus *spp.,* Enterobacteriaceae*,* Pseudomonas aeruginosa*, and others [5, 8]. As reported by Cotton et al. numerous pathogenic bacteria, including* Salmonella *spp.,* Shigella *spp.,* Campylobacter* spp.,* Pseudomonas aeruginosa*, and* Klebsiella pneumoniae* have been isolated from cockroaches [4] and these insects greatly contribute to food-borne disease outbreaks [9]. As a result insects like cockroaches collected in hospitals and households have been found to harbor multidrug resistant (MDR) bacteria and hospital cockroaches with drug-resistant* Klebsiella* spp. have been suggested to play a role in the epidemiology of nosocomial infections [5, 8]. In addition, a neonatal unit infested with cockroaches suffered an outbreak of nosocomial disease due to extended-spectrum *β*-lactamase-producing* K. pneumoniae* [4].

Even though cockroaches are medically important as many of infectious diseases have been associated with them, the public health importance of this vector has not been well documented in the study area. Therefore, the aim of the study was to isolate pathogenic bacteria from cockroaches and to determine their antimicrobial susceptibility patterns.

## 2. Methods

### 2.1. Study Area and Data Collection

A total of 60 cockroaches were collected from January to May 2014 in Gondar town. Thirty of them were obtained from various types of wards in University of Gondar hospital. The remaining 30 s were trapped from nonhospital environments including different parts of the house (kitchens, bathrooms, and toilets). Cockroaches were collected using sterile test tubes and transported to the microbiology laboratory for bacteriological analysis within two hours of collection. Species identification was done in accordance with a standard taxonomic key.

### 2.2. Culture and Identification of Bacterial Isolates

Cockroaches were immobilized by frigidity at 0°C for 5 minutes. Sterile normal saline (5 mL) was added to each test tube and cockroaches were vigorously washed and transferred to secondary sterile test tubes. A loop full of each suspension was cultured on MacConkey agar (MAC), blood agar plate, and chocolate agar plate and left for 24 hours. Additionally, isolation and identification of microorganisms from internal surfaces of cockroaches were also performed following standard procedure. After subsequent washing and decontamination by using 70% alcohol, the gut of the cockroaches was dissected out and macerated aseptically in a sterile pestle and mortar. Similarly, each suspension of cockroaches was cultured on the above-mentioned culture media for bacteriological investigation [10].

A pure colony of bacterial isolates was preliminary characterized by colony morphology and Gram-staining procedure. A standard biochemical procedure was used for full identification of Gram positive and Gram negative bacteria [11].

### 2.3. Antimicrobial Susceptibility Testing

Antimicrobial susceptibility testing was performed for bacterial isolates by using agar diffusion method on Mueller-Hinton agar (Oxoid). Bacterial inoculum was prepared by suspending the freshly grown bacteria in 4-5 mL sterile nutrient broth and the turbidity was adjusted to that of a 0.5 McFarland standard. The antimicrobial susceptibility testing was performed against the following disks (Oxoid, UK): gentamicin (GEN; 10 *μ*g), chloramphenicol (C; 30 *μ*g), ciprofloxacin (CIP; 5 *μ*g), erythromycin (15 *μ*g), methicillin (MET; 5 *μ*g), penicillin (PEN; 10 units), amoxicillin-clavulanate (AMC; 30 *μ*g), vancomycin (VAN; 30 *μ*g), cotrimoxazole (SXT; 25 *μ*g), tetracycline (TE; 30 *μ*g), ceftriaxone (CTR; 30 *μ*g), and ceftazidime (CAZ; 30 *μ*g). After overnight incubation, the diameter of the zone of inhibition around the disc was measured and interpreted as susceptible, intermediate, and resistant according to National Committee for Clinical Laboratory Standards, and the isolates showing resistance to two or more different classes of antibiotics are considered as multidrug resistant (MDR) strains [12].

### 2.4. Quality Control

Culture media were tested for sterility and performance. Standard strains of* E. coli* ATCC 25922 and* S. aureus* ATCC 25923 were used during culture and antimicrobial susceptibility testing.

### 2.5. Data Analysis

Data were entered and analyzed using SPSS version 20 statistical software and presented through tables and graph. Associations were measured using Pearson's chi-square test. *P* values <0.05 were considered statistically significant.

### 2.6. Ethical Considerations

An ethical approval was obtained from ethical clearance committee of the University of Gondar. Informed written consent was also obtained from each study site (from hospital administration or household owner). All information obtained at each course of the study was kept confidential. The findings of the study were communicated and oriented for their better management of their house or wards to avoid the vectors.

## 3. Results

### 3.1. Bacterial Isolates

A total of sixty cockroaches were trapped from hospital and nonhospital environment. One hundred eighty-one bacterial species were isolated from 100% examined cockroaches.* Klebsiella pneumoniae* (17.7%) was the leading isolate from external and internal surfaces of cockroaches followed by* E. coli* (16%) and* Citrobacter* species (15%). Besides,* K. pneumoniae* was also the commonest isolate from cockroaches in hospital environment, whereas* E. coli* and* Citrobacter* species were predominantly isolated from nonhospital cockroaches (Table 1).

### 3.2. Antibiotic Susceptibility Pattern

All of the isolated bacterial isolates were tested to determine their antibiotic susceptibility pattern. The overall resistance rates of isolates are presented in Table 2; Gram positive bacteria such as* S. aureus* and coagulase negative staphylococci (CNS) species showed 100% resistance rate for penicillin; fortunately no resistance rate was observed to methicillin and vancomycin. Comparatively, all isolates demonstrated high resistance rates to cotrimoxazole followed by amoxicillin-clavulanate and tetracycline, whereas low resistance rates were noted to ciprofloxacin. Besides, species specific resistance rates are also indicated in Table 2; more than 50% of* Shigella* species were found to be resistant to cotrimoxazole, chloramphenicol, ceftazidime, tetracycline, and ceftriaxone and over 74% of* Salmonella* species were also resistant to gentamycin, cotrimoxazole, tetracycline, chloramphenicol, ceftriaxone, and ceftazidime.

**Figure 1:**
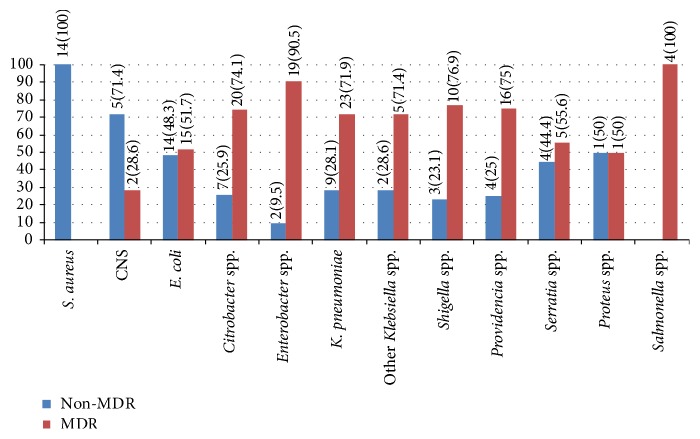
Proportion of MDR and non-MDR isolates identified from cockroaches, Gondar, 2014.* S. aureus*:* Staphylococcus aureus*; CNS: coagulase negative staphylococci;* E. coli*:* Escherichia coli*;* Enterobacter* species:* Enterobacter cloacae* and* aerogenes*;* K. pneumoniae*:* Klebsiella pneumoniae*; other* Klebsiella* species:* Klebsiella ozaenae* and* rhinoscleromatis*. Non-MDR: nonmultidrug resistant and MDR: multidrug resistant.

Moreover, the isolates were also assessed for MDR pattern, 116 (64.1%) of them identified as resistance to two or more classes of antibiotics. As summarized in Figure 1 the proportion of MDR bacteria within species level has been determined; thus all* Salmonella* species were found to be MDR isolates followed by* Enterobacter* (90.5%) and* Shigella* species (76.9%).  Table 3 indicated that the rate of MDR isolates identified from hospital collected cockroaches, 67% (59/88), was higher than nonhospital environment, 61.3% (57/93), but it was not statistically significant (67% versus 61.3%; *P* = 0.420). On the other hand, the rate of MDR* E. coli* in nonhospital cockroaches was significantly higher than hospital environment (66.7% versus 27.3%; *P* = 0.039).

## 4. Discussion

Cockroaches are common in many of human habitations, particularly in place where food is stored, processed, prepared, or served. Apart from that, they are also frequently detected in hospital environments, such as wards, operational rooms, area of intensive care units, and laboratory rooms [13, 14]. Indeed, cockroaches are found everywhere and possess nocturnal and omnivorous features; these characteristics make them the ideal carriers of pathogenic microorganisms including bacteria, protozoa, helminthes, fungus, and virus [15, 16]. It is well indicated that cockroaches are known to harbor pathogens, which can cause potentially devastating diseases, such as gastroenteritis, typhoid, and diarrheal syndromes [6]. According to findings, cockroaches are the main source of bacterial pathogens and they are also associated with multiple drug resistant strains. Therefore they have a great impact on the spread of diseases and dispersal of MDR bacterial strains [4, 5, 14, 17].

The present study demonstrated that cockroaches are the potential source of pathogenic bacteria. Hence, 181 bacterial species from 12 different genera were identified; predominantly the isolates were* Enterobacteriaceae*, 160/181 (88.4%), and the rest were Gram positive bacteria (*S. aureus* and CNS), 21/181 (11.6%). Of the isolates,* K. pneumoniae* was the leading isolate from external and internal surfaces of cockroaches followed by* E. coli *and* Citrobacter *spp. It is known that isolates are the main causes of diverse types of nosocomial and community acquired infections, notably pneumonia, urinary tract infection, respiratory tract infection, skin infections, septicemia, and gastroenteritis [18]. Likewise previous reports have also indicated that the above-mentioned bacteria pathogens were the common isolates from cockroaches [10, 19–21].

This study has also explored antibiotic resistance patterns of isolates and it was surprising that high resistance rates were observed against some of the antibiotics. Among antibiotics tested for all isolates, more than half of isolates were found to be resistant to cotrimoxazole, and especially Gram positive bacterial isolates have showed complete resistance to penicillin. However, ciprofloxacin was relatively the most powerful antibiotic against the isolates. Even though, antibiotics are not normally applied on cockroaches, but it is known that high resistance rates were reported among pathogens associated foods [22, 23]. In fact, a great association between cockroaches and foods could be the probable reason for isolation of resistant strains from cockroaches.

Surprisingly, studies showed that MDR strains have been also demonstrated from cockroaches. In addition, this finding revealed that 116 (64.1%) of isolates were found to be MDR strains. It is known that MDR strain could arise due to accumulation of resistant genes in a single bacterial cell or expression of genes that code for multidrug efflux pumps, extruding a wide range of drugs [24]. Since cockroaches are the main source of bacterial pathogens and antibiotic resistance strains [10, 25]. Effective prevention and control are necessary to reduce nosocomial and food-borne bacterial infections. Various activities should be implemented for instant use of tightly fitted food containers, discarding cardboards, and general sanitation of equipment and facilities to remove all food debris and dirt. Sometimes pest controls methods can be also employed using insecticides (boric acid), which are applied to the resting and hiding places as residual sprays and insecticidal dusts [26, 27].

## 5. Conclusion

A large number of bacterial species were recovered from cockroaches. Enterobacteria were the principal isolates. Relatively, high resistance rates were noted in cotrimoxazole, but ciprofloxacin was the most effective antibiotic against isolates. Gram positive bacteria especially (*S. aureus* and CNS) have showed extreme resistance to penicillin. Moreover,* Salmonella*,* Shigella*, and* Enterobacter* spp. were found to be predominant MDR isolates. Since cockroaches are the potential source of bacterial pathogen and MDR stains and, therefore, appropriate preventive, and control measures are suggested to reduce cockroach associated infections.

## Figures and Tables

**Table 1:** Bacterial isolates identified from external and internal surfaces of cockroaches, Gondar, 2014.

Bacterial isolate	External surfaces		Internal surfaces		Total
	Hosp	Non-hosp	Hosp	Non-hosp	
*S. aureus*	9 (69.2)	4 (30.8)	1 (100)	0	14 (7.7)
CNS	3 (42.9)	4 (57.1)	0	0	7 (3.9)
*E. coli*	6 (37.5)	10 (62.5)	5 (38.5)	8 (61.5)	29 (16)
*Citrobacter* spp.	5 (33.3)	10 (66.7)	4 (33.3)	8 (66.7)	27 (15)
*Enterobacter* spp.	5 (45.5)	6 (54.5)	4 (40)	6 (60)	21 (11.6)
*K. pneumoniae*	13 (76.5)	4 (23.5)	12 (80)	3 (20)	32 (17.7)
Other *Klebsiella* spp.	0	6 (100)	0	1 (100)	7 (3.9)
*Shigella* spp.	6 (75)	2 (25)	3 (60)	2 (40)	13 (7.2)
*Providencia* spp.	5 (62.5)	3 (37.5)	6 (75)	2 (25)	16 (8.8)
*Serratia* spp.	1 (14.3)	6 (85.7)	1 (50)	1 (50)	9 (5)
*Proteus* spp.	0	1 (100)	0	1 (100)	2 (1)
*Salmonella* spp.	1 (100)	0	3 (100)	0	4 (2.2)

*Total*	54 (49.1)	56 (50.9)	39 (54.9)	32 (45.1)	181 (100)

*S. aureus*, *Staphylococcus aureus*; CNS, coagulase negative staphylococci; *E. coli*, *Escherichia coli*; *Enterobacter* species, *Enterobacter cloacae* and *aerogenes*; *K. pneumonia*, *Klebsiella pneumoniae*; other *Klebsiella* species, *Klebsiella ozaenae* and *rhinoscleromatis*; Hosp, hospital environment; Non-hosp, nonhospital environment.

**Table 2:** Resistance rates of bacterial isolates identified from cockroaches, Gondar, 2014.

Bacterial isolates	Antibiotics												
	GEN	SXT	TE	C	AMC	CIP	NA	CTR	CAZ	MET	VAN	PEN	ERY
*S. aureus *	0	0	0	0	0	0	NT	NT	NT	0	0	14 (100)	0
CNS	0	2 (28.6)	1 (14.3)	0	0	0	NT	NT	NT	0	0	7 (100)	1 (14.3)
*E. coli *	3 (10.3)	4 (13.8)	4 (13.8)	2 (6.9)	4 (13.8)	0	3 (10.3)	3 (10.3)	4 (13.8)	NT	NT	NT	NT
*Citrobacte*r spp.	2 (7.4)	8 (29.6)	8 (29.6)	6 (22.2)	11 (40.7)	0	1 (3.7)	6 (22.2)	9 (33.3)	NT	NT	NT	NT
*Enterobacter* spp.	6 (28.6)	8 (38.1)	5 (23.8)	5 (23.8)	9 (42.9)	0	1 (4.8)	5 (23.8)	7 (33.3)	NT	NT	NT	NT
*K. pneumoniae *	11 (34.4)	17 (53.1)	14 (43.8)	5 (15.6)	NT	0	0	10 (31.3)	13 (40.6)	NT	NT	NT	NT
Other *Klebsiella* spp.	0	0	0	1 (14.3)	3 (42.9)	0	0	0	0	NT	NT	NT	NT
*Shigella* spp.	6 (46.2)	10 (76.9)	7 (53.8)	9 (69.2)	2 (15.4)	0	1 (7.7)	7 (53.8)	9 (69.2)	NT	NT	NT	NT
*Providencia* spp.	6 (37.5)	6 (37.5)	NT	0	11 (68.8)	2 (12.5)	2 (12.5)	4 (25)	4 (25)	NT	NT	NT	NT
*Serratia* spp.	0	1 (11.1)	0	0	1 (11.1)	0	0	1 (11.1)	1 (11.1)	NT	NT	NT	NT
*Proteus* spp.	0	0	NT	2 (100)	0	0	0	0	0	NT	NT	NT	NT
*Salmonella* spp.	3 (75)	4 (100)	3 (75)	3 (75)	0	0	0	3 (75%)	3 (75%)	NT	NT	NT	NT

Total (*N* = 181)	37 (20.4)	60 (33.1)	42^*∗*^ (25.8)	33 (18.2)	41^*∗*^ (27.5)	2 (1.1)	8^*∗*^ (5)	39^*∗*^ (24.4)	50^*∗*^ (31.3)	0	0	21^*∗*^ (100)	1^*∗*^ (4.8)

NT, not tested; ^*∗*^NT isolates were not included in the denominator; GEN, gentamycin; SXT, cotrimoxazole; TE, tetracycline; C, chloramphenicol; AMC, amoxicillin-clavulanic acid; CIP, ciprofloxacin; NA, nalidixic acid; CTR, ceftriaxone; CAZ, ceftazidime; MET, methicillin; VAN, vancomycin; PEN, penicillin; ERY, erythromycin; *S. aureus*, *Staphylococcus aureus*; CNS, coagulase negative staphylococci; *E. coli*, *Escherichia coli*; *Enterobacter* species, *Enterobacter cloacae* and *aerogenes*; *K. pneumoniae*, *Klebsiella pneumoniae*; other *Klebsiella* species, *Klebsiella ozaenae* and *rhinoscleromatis*.

**Table 3:** The proportion of MDR and non-MDR isolates in cockroaches from hospital and nonhospital environment, Gondar, 2014.

Bacterial isolate	Site of collection	Non-MDR	MDR	*P* value
*S. aureus*	Hosp	10 (100)	0	—
	Non-hosp	4 (100)	0	

CNS	Hosp	1 (33.3)	2 (66.7)	0.053
	Non-hosp	4 (100)	0	

*E. coli*	Hosp	8 (72.7)	3 (27.3)	**0.039**
	Non-hosp	6 (33.3)	12 (66.7)	

*Citrobacter*	Hosp	1 (11.1)	8 (88.9)	0.214
	Non-hosp	6 (33.3)	12 (66.7)	

*Enterobacter* spp.	Hosp	2 (22.2)	7 (77.8)	0.086
	Non-hosp	0	12 (100)	

*K. pneumoniae*	Hosp	9 (36)	16 (64)	0.061
	Non-hosp	0	7 (100)	

*Klebsiella* spp.	Non-hosp	2 (28.6)	5 (71.4)	—

*Shigella* spp.	Hosp	1 (11.1)	8 (88.9)	0.125
	Non-hosp	2 (50)	2 (50)	

*Providencia* spp.	Hosp	3 (27.3)	8 (72.7)	0.755
	Non-hosp	1 (20)	4 (80)	

*Serratia* spp.	Hosp	1 (50)	1 (50)	0.858
	Non-hosp	3 (42.9)	4 (57.1)	

*Proteus* spp.	Non-hosp	1 (50)	1 (50)	—

*Salmonella* spp.	Hosp	0	4 (100)	—

Total isolates	Non-hosp	36 (38.7)	57 (61.3)	0.420
	Hosp	29 (33)	59 (67)	

*S. aureus*, *Staphylococcus aureus*; CNS, coagulase negative *Staphylococci*; *E. coli*, *Escherichia coli*; *Enterobacter* species, *Enterobacter cloacae* and *aerogenes*; *K. pneumoniae*, *Klebsiella pneumoniae*; other *Klebsiella* species, *Klebsiella ozaenae* and *rhinoscleromatis*; Non-MDR, nonmultidrug resistant; MDR, multidrug resistant; Hosp, hospital environment; Non-hosp, nonhospital environment.

## Abbreviations

CNS: Coagulase negative staphylococci
MDR: Multidrug resistant.
